# Climate change, household vulnerability and smart agriculture: The case of two South African provinces

**DOI:** 10.4102/jamba.v8i2.182

**Published:** 2016-01-13

**Authors:** Mkhululi Ncube, Nomonde Madubula, Hlami Ngwenya, Nkulumo Zinyengere, Leocadia Zhou, Joseph Francis, Talentus Mthunzi, Crespo Olivier, Tshilidzi Madzivhandila

**Affiliations:** 1 Financial and Fiscal Commission, Johannesburg, South Africa; 2 Food, Agriculture and Natural Resources Policy Analysis Network, Pretoria, South Africa; 3 Climate Systems Analysis Group, University of Cape Town, South Africa; 4 Risk and Vulnerability Science Centre, University of Fort Hare, South Africa; 5 Institute for Rural Development, University of Venda, South Africa

## Abstract

The impact of climate-change disasters poses significant challenges for South Africa, especially for vulnerable rural households. In South Africa, the impact of climate change at the local level, especially in rural areas, is not well known. Rural households are generally poor and lack resources to adapt to and mitigate the impact of climate change, but the extent of their vulnerability is largely not understood. This study looked at the micro-level impact of climate change, evaluated household vulnerability and assessed alternative adaptation strategies in rural areas. The results indicate that climate change will hit crop yields hard and that households with less capital are most vulnerable. These households consist of the elderly and households headed by females. Households that receive remittances or extension services or participate in formal savings schemes in villages are less vulnerable. The results suggest that households need to move towards climate-smart agriculture, which combines adaptation, mitigation and productivity growth.

## Introduction

Developing countries are increasingly vulnerable to disasters. In South Africa, the millions of people living in rural areas are amongst the poorest and the most vulnerable and have a low capacity for resilience with which to cope with disaster risks. Current interventions fail to recognise the different types of impact of climate-change disasters on people’s livelihoods, especially in rural households. Identifying those households and livelihoods that are vulnerable to climate change has become a key input for targeting, formulating, monitoring and evaluating adaptation policies.

Climate change is a global externality that negatively affects households, communities and the broader economy. The potential of climate change to destabilise economies and public finances is real and can no longer be ignored. Climate change is associated with many of the natural disasters in South Africa and can lead to widespread food and water insecurity. A heavy dependence on climate-sensitive economic sectors, in particular agriculture, makes South Africa particularly vulnerable to climate change. The effect of climate change on agricultural output will directly affect rural communities through reduced income and employment, and it will have a knock-on effect on both the rural economies and the food-security nexus (FFC 2012). Rural households are more vulnerable because they lack the means for adaptation. As (human, financial and physical) resources are limited, it is important that these scarce resources are targeted at the most vulnerable communities.

South Africa’s disasters and its food and water insecurity are often analysed at the aggregate level whereas identifying vulnerable households is critical in order to formulate well-targeted adaptation and mitigation policies and strategies. In South Africa, a number of studies have analysed vulnerability at the household level but have scarcely examined the dimensions of vulnerability within these households. As acknowledged by the National Disaster Management Framework (Government of South Africa 2005), the lack of such data is one of the challenges hampering the effectiveness of disaster-risk management. When a climate-change disaster strikes, the first point of call is at the local level, which means that it is important to know who is vulnerable – not only by area but also by household and gender.

The following research questions are the linchpin of this study: (1) What is or will be the impact of climate change at the household level? (2) Who is vulnerable to disasters related to climate change? (3) How vulnerable are households, and what are the causes of their vulnerability? (4) How can households adapt to disasters related to climate change, and what are the costs and benefits of different adaptation strategies?

To answer these questions, this study (1) models the impact of climate change at local level; (2) uses the Household Vulnerability Index (HVI) to evaluate the vulnerability of rural households to natural disasters, food and water insecurities; (3) establishes the costs and benefits of different rural adaptation strategies and (4) provides recommendations on the fiscal-policy measures and instruments that can be used to improve the resilience of households.

After describing the methodology in the next section, the study results are analysed, followed by the conclusion and policy recommendations.

## Methodology

**FIGURE 1:**
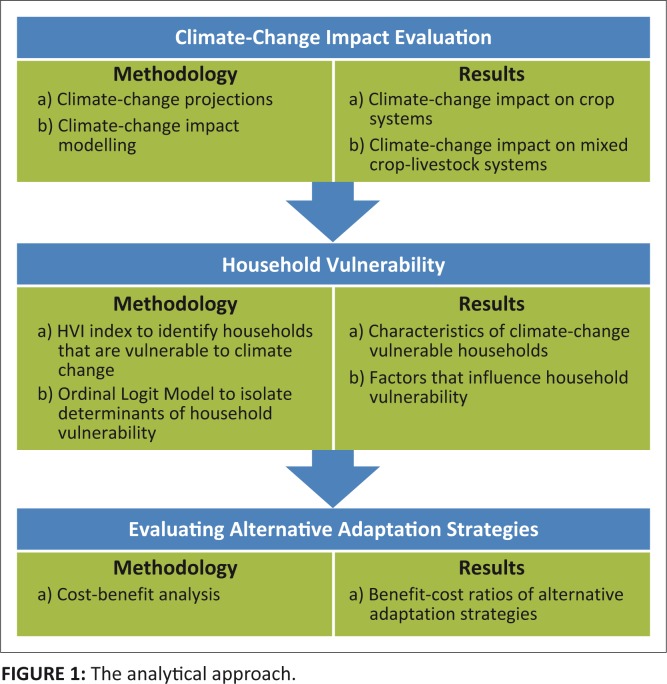
The analytical approach.

Figure 1 describes the analytical approach. The different methodologies are explained in the following sub-sections.

### Methodology for evaluating the impact of climate change

#### Historical climate and future projections

The process-based crop model of the Decision Support System for Agro-technology Transfer (DSSAT) is used to assess the agricultural response to future climate change. The model was calibrated and validated at each location and is used to simulate the maize-cropping potential under projected climate-change scenarios. Outcomes simulated under two General Climate Models (GCMs), issued from the most recent Comprehensive Model Inter-comparison Project (CMIP) Phase 5, were used. Historical records of the climate were collected for each location from the Climate System Analysis Group’s (CSAG) public records.^1^ CMIP5’s future scenarios provide large-scale projections running from 1960 to 2100. At each study location, the scenarios computed by the selected GCMs were downscaled to station level.

The focus was on the impact of climate change on maize-crop systems in the early century (2010–2040) and mid-century (2040–2070), relative to a baseline (1980–2010). A historical daily weather dataset (1980–2010) was generated, using the best available data for geographical proximity, data length and quality. Daily minimum and maximum temperatures and rainfall were used. For Alice (Eastern Cape), climate characterisation was done based on the Fort Beaufort station’s historical daily records whilst for Lambani (Limpopo) the Punda Maria station’s historical daily records were used.

Management strategies used by farmers in each study location were simulated and remained unchanged between baseline and future periods so that only the effect of climate change on maize yields was represented. To simulate the impact of climate change at the community scale, the crop model used local, experimental maize trials.

Process-based crop models require complete daily datasets to run, but observations inevitably include missing data. The gaps were filled with the Modern Era Retrospective-Analysis for Research and Applications (MERRA). Each station consisted of an observed and filled-in dataset, referred to as historical climate. Potential evapotranspiration was not available for either station and was thus estimated at a daily timescale, using temperature and rainfall from the historical climate records as well as latitude and altitude (see Allen *et al*. 1998).

For future projections, the latest GCMs archived in the CMIP5 were used. The CMIP5 version provides continuous daily minimum and maximum temperatures and rainfall data from 1960 to 2100 (2099 in some cases). In order better to suit the field-scale nature of the crop model, daily climate records (un-filled) of minimum and maximum temperatures and precipitation were used to downscale the GCMs large-scale cells to station scale, based on a self-organising map methodology (Hewitson & Crane 2006). Each GCM simulation was downscaled to a climate station in the study area, namely Punda Maria (Vhembe District – Limpopo) and Fort Beaufort (Nkonkobe District – Eastern Cape). This gave a local representation of the large-scale circulation as projected by GCMs. CSAG provided access to projections from 10 GCMs per location under two Representative Concentration Pathways (RCPs): RCP4.5 and RCP8.5 as a representation of median and high CO_2_ concentration trajectories into the future (IPCC 2013).

Given the time and resource constraints of the project, only two GCMs and two RCPs were used for the crop-modelling simulations. The GCMs from the Centre National de Recherches Météorologiques (CNRM) and the Flexible Global Ocean-Atmosphere-Land System (FGOALS) were selected at both locations to represent two different scenarios of projected climate conditions. A baseline period (1980–2010) and two future-time periods were used, namely early-century (2010–2040) and mid-century periods (2040–2070).

The projections for crop-model simulations for the early (2010–2040) and mid-21st century (2040–2070) were run over a 31-year period. To emphasise the time-period climatology, only a 20-year window was selected and presented, namely 2015–2035 for early century and 2045–2065 for mid-century. Although only two GCMs were used for crop-model simulations, all GCM projections provided by CSAG are presented.

#### Calibration and validation

Calibration and validation was performed using experimental trials obtained from published research papers. The DSSAT (version 4.5) model (Jones *et al*. 2003) was calibrated and validated for maize at both study locations with experiments that consisted of a range of treatments that varied by location and season. Model calibration was achieved through tuning phenology and growth coefficients of a crop variety by minimising the differences between observed and simulated crop yields for a season’s simulation trial. Data constraints meant that only one season was used for calibration at each research site. The model was run three months prior to planting date in order to estimate initial soil water. Crop varieties calibrated were identified from reported trials and the cultivars identified and used per location. The coefficients were adjusted from varieties already found in the DSSAT database that were within a similar growth category (early maturing, medium maturing, et cetera). The established cultivar parameters were applied for model validation. Soil types were identified from published reports and used to identify similar soils within the DSSAT database.

Lambani (Vhembe District, Limpopo) has a short growing season whereas Alice (Eastern Cape) has a long growing season. Therefore, a short-season maize variety (SNK 2147) was set up for local conditions in Lambani, and a long-season maize cultivar (PAN6777) was set up to simulate maize yields in Alice. For Lambani, the experiment was carried out over two seasons (Odhiambo 2011): the 2006–2007 season was used for calibration and the 2007–2008 season for validation. The crop model (DSSAT) was able to simulate maize yields in response to climate and agronomic management and to simulate observed yield within a 5% relative difference (RD). In Alice, the 200910 cropping season was used for calibration (Fanadzo, Chiduza & Mnkeni 2010) whilst validation was performed over two seasons: 2005–2006 and 2007–2008 (Fanadzo, Chiduza & Mnkeni 2009). DSSAT was found to be suitable for simulating maize yields in response to climate and agronomic management. Maize yields were simulated within a 7% RD. The results are shown in Table 1.

**TABLE 1:** Simulated and observed yields per season and management conditions.

Location	Seasons	Planting dates	Fertiliser kg N/ha	Planting density plants/ha	Observed kg/ha	Simulated kg/ha	RD %
Alice	2009/10	10/11	60	40 000	4507	4527	+0.44
	2005/06	10/11	60	40 000	3853	3611	+6.28
	2005/06	10/12	60	40 000	4286	4000	+6.70
	2007/08	Mid Nov	60	41 125	3800	3985	+4.87
Lambani	2006/07	15/11	75	44 400	4900	4861	−0.82
	2007/08	01/11	75	44 400	7400	7045	−4.80

RD, relative difference.

**FIGURE 2:**
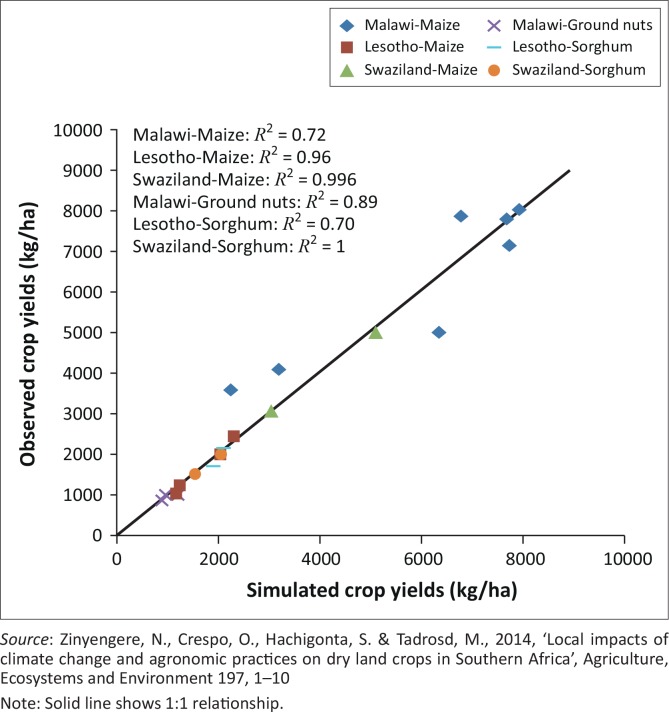
Relationship between observed and simulated crop yields Malawi and Swaziland.

The model performs well, given the available data. Additional data would likely result in higher confidence in the ability of the crop model to simulate yields under specific conditions. Further confidence was obtained from expert consultations during site visits and from previous work that showed a strong relationship between simulated and observed yields in various agro-ecological environments and crops in Swaziland, Lesotho and Malawi, as shown in Figure 2 (Zinyengere, Crespo & Hachigonta 2013). However, despite considerable confidence in the model, decision makers need to take spatial and temporal limitations into consideration when interpreting the numerical outcomes.

### Methodology for assessing household vulnerability

The paper’s second objective is to assess the vulnerability of households to disasters related to climate change. The concept of vulnerability is relative and dynamic. Vulnerability can be viewed as a loss in welfare because of the adverse state of nature (for example, the impact of climate change). It can be defined as the diminished capacity of an individual or group to anticipate, cope with, resist and recover from the impact of a natural or man-made hazard. The UK’s Department for International Development (DFID) defines vulnerability as the susceptibility of individuals, households or communities to become poor or poorer as a result of events or processes that affect their livelihood systems (DFID 2000). In other words, vulnerability refers to the extent to which one is prone, at risk or likely to be food insecure. Vulnerability is most often associated with poverty but can also arise when people are isolated, insecure and defenceless in the face of risk, shock or stress (Birkman 2006). Distinguishing between the external and internal is the starting point for disaggregating vulnerability. Vulnerability has two sides, namely an external side with risks, shocks and stress to which an individual is subject and an internal side or being defenceless, meaning a lack of means with which to cope when facing loss. Loss can take many forms such as becoming or being physically weaker, economically improvised, socially dependent, humiliated or psychologically harmed.

The general welfare of a household can at the level of consumption, utility or poverty. Econometric approaches for estimating household vulnerability rely on socio-economic survey data (Deressa, Hassan & Ringler 2009) and include vulnerability as uninsured exposure to risk (VER), low expected utility (VEU) and expected poverty (VEP) (Hoddinott & Quisumbing 2003). The three methods are used to construct measures of welfare loss as a result of climatic shocks. The lack of consensus on how to estimate household vulnerability is highlighted through the difficulties associated with VEP, VER and VEU.

VER is based on an assessment before and after the fact, considering the extent to which a negative shock causes welfare loss. Panel datasets are used to quantify the impact of the shock on consumption. Without risk-management tools, shocks will always impose welfare loss that manifests in a reduction in consumption. Moreover, the lack of panel data results in estimates of impact (particularly from cross-sectional data) that are often biased and, therefore, inconclusive (Skoufias 2003).

Focusing on the VEU method, Ligon and Schechter (2003) describe vulnerability as follows:

… the difference between the utility derived from some level of certainty-equivalent consumption … at and above which the household would not be considered vulnerable, and the expected utility of consumption. (p. 45)

The VEP method views vulnerability as the likelihood of a household becoming poor in future or the prospect of its continuing to be poor (Christiaensen & Subbarao 2004). This method regards vulnerability as expected poverty in the event of a shock with consumption or income used as the welfare indicator (Chaudhuri, Jalan & Suryahadi 2002).

#### Food, Agriculture and Natural Resources Policy Analysis and Network’s Household Vulnerability Index

**FIGURE 3:**
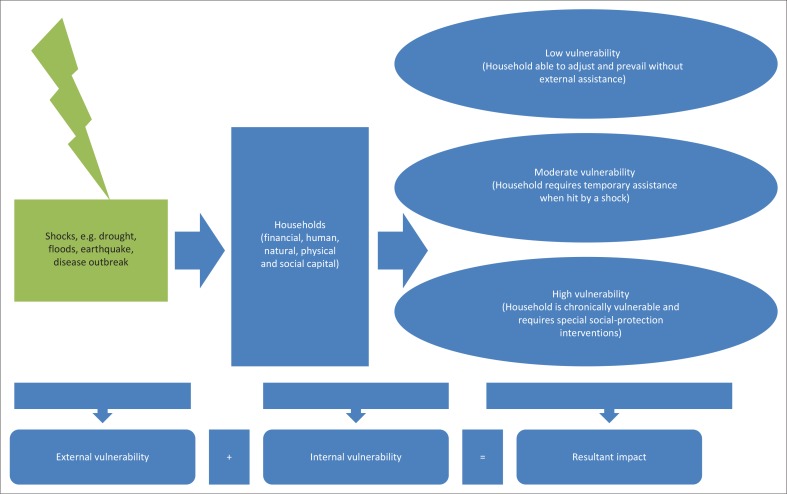
Conceptual framework for household-vulnerability assessment.

This study uses Food, Agriculture and Natural Resources Policy Analysis and Network (FANRPAN)’s HIV (FANRPAN 2003–2007) to understand the vulnerability of households to climate-change disasters (see Figure 3). The HVI assesses ‘external’ vulnerability, introduced by a defined shock or shocks, and ‘internal’ vulnerability, a household’s inability to withstand shocks in general. It is based on the Sustainable Livelihoods Framework (SLF) developed by DFID (DFID 2000). SLF analyses the livelihoods of the poor, using the lens of financial, human, natural, physical and social capital. It uses fuzzy logic to assess a household’s access to (1) natural assets such as land, soil and water; (2) physical assets such as livestock and equipment; (3) financial assets such as savings, salaries, remittances or pensions; (4) human-capital assets such as farm labour, gender composition and dependents; and (5) social assets such as information, community support, extended families and formal or informal social-welfare support. More than 15 variables (called dimensions) are assessed together, and a statistical score is calculated for each household. Households are categorised into the low, medium and high vulnerability:

- Low vulnerability: households that will adjust to the given shock (for example by using their capital assets) and do not need significant external support.
- Moderate vulnerability: households that require some level of external assistance to overcome the given shock.
- High vulnerability: households that need expert assistance to recover from the impact of shocks. These households can be likened to intensive-care situations in hospitals. High-vulnerability households may be welfare cases, on a downwards spiral, and may eventually cease to exist without timely and appropriate support.

FANRPAN’s HVI was tested by investigating the impact of HIV and AIDS on agriculture and food security in seven countries: Botswana, Lesotho, Namibia, South Africa, Swaziland, Zambia and Zimbabwe. A re-run study was conducted in three countries to refine the tool: Lesotho, Swaziland and Zimbabwe. Based on the different vulnerability levels, as elaborated above, specific relief or development packages were recommended to assist the affected households in overcoming their vulnerability. All the studies confirm that the HVI is a useful tool for planning and policy development because it provides a yardstick for determining the extent of certain social challenges, making it possible to measure the progress of a particular development strategy or policy on a time series and linear scale.

The HVI may be a robust and rigorous tool, implemented for over 18 000 households in Southern Africa, but has limitations regarding the understanding of household vulnerability to climate-change disasters. Firstly, using the HVI for the cost-benefit analysis (CBA) of the proposed adaptation measures incurs some challenges, primarily because the HVI does not include costs of assets and services.^2^ Secondly, the HVI does not account for gender dimensions of vulnerability, and yet women are amongst the poorest of the poor, often unable to access better opportunities and prevented from being economically independent by the patriarchal setup. This study will improve on previous HVI work by focusing on three components of vulnerability (exposure, sensitivity and adaptive capacity), integrating gender and including CBA (see section 2.3).

### Isolating the determinants of household vulnerability

**The ordinal logit model:** The ordered probit model of Aitchison and Silvey (1957) and McKelvey and Zavoina (1975) was used in this study rather than a multinomial regression. An ordinary linear regression is inappropriate for ordinal responses because of the non-interval nature of the dependent variable (Long 1997) whilst multinomial logit models would fail to account for the ordinal nature of the dependent variable and thus not employ all of the information available in that variable.

**Model specification:** The ordered probit model is usually motivated in a latent (unobserved) variables framework. The general specification is:

$$y_{i}^{*}=X_{i}\beta +\varepsilon_{i}$$

where y_i_^*^ is the latent variable measuring the *i*th vulnerability household; *X_i_* is the is a (*k* × 1) vector of observed non-random explanatory variables; *β* is a (*k* × 1) vector of unknown parameters and *ε_i_* is the random error term, which is assumed to be normally distributed with zero mean and unit variance. *y_i_* is determined from the model as follows:

$$y_{i}=\{\begin{array}{c} 1,\text{if}\mathrm{-\infty}\leq {\text{y}}_{\text{i}}^{*}\leq \mu_{1}\\ 2,\text{if}\mu_{1}\leq {\text{y}}_{\text{i}}^{*}\leq \mu_{2}\\ 3,\text{if}\mu_{2}\leq {\text{y}}_{\text{i}}^{*}\leq \infty \end{array}$$

where the threshold values *μ*_1_ and *μ*_2_ are unknown parameters to be estimated.

The estimates of the ordinal approach tend to be heteroskedastic, given the nature of the data used. Equation (2) was estimated using the maximum likelihood method, taking the two regimes jointly (Wooldridge 2002). Heteroscedasticity was, however, significant in the cross-sectional data as indicated by the Breusch-Pagan/Cook-Weisberg test (*p*-value < 0.0002). To correct this problem (and thus improve the efficiency of the estimates), the study used robust standard errors. In addition, the data were tested for proportionality, which is a fundamental assumption for an ordinal regression model. The Brant Test indicated that the assumption of proportional odds (which means that each independent variable has an identical effect at each cumulative split of the ordinal dependent variable) still holds for 14 of the 17 independent variables.

### Methodology for evaluating alternative adaptation strategies

#### Cost-benefit analysis

The paper’s third objective is to assess costs and benefits in order to isolate the best adaptation strategies for rural communities. CBA is an important and effective management tool for analysing any type of investment (Chae 2010), in particular in the initial life cycle of the investment (Noleppa 2013). Adaptation is an investment that should be done in a cost-effective way. This requires a thorough understanding of the size and regional distribution of damage, in addition to a precise assessment of the cost or effectiveness of alternative actions and their strategic complementarity or trade-offs (Carraro, Bossello & De Cian 2009).

**FIGURE 4:**
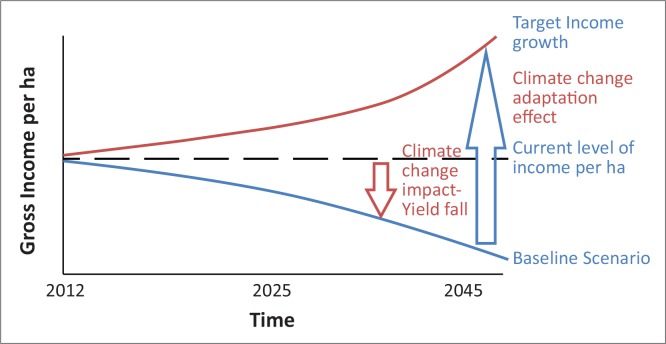
Impact of climate change on gross income per hectare (2012–2042).

Figure 4 shows the general rule relating to the impact of climate change on current production, including how adaptation might bring about the required growth in agriculture. Adaptation strategies vary both in terms of efficiency and targeted climatic risk (Carraro *et al*. 1999), but adopting and accurately applying appropriate adaptation strategies can assist in the efficient allocation of resources.

CBA is often used to assess adaptation options when efficiency is the only decision-making criterion. It involves calculating and comparing all the costs and benefits, expressed in monetary terms. A comparison of expected costs and benefits help to inform decision makers about the likely efficiency of an adaptation investment (Chae 2010). In this respect, CBA provides a basis for prioritising possible adaptation measures.

**FIGURE 5:**
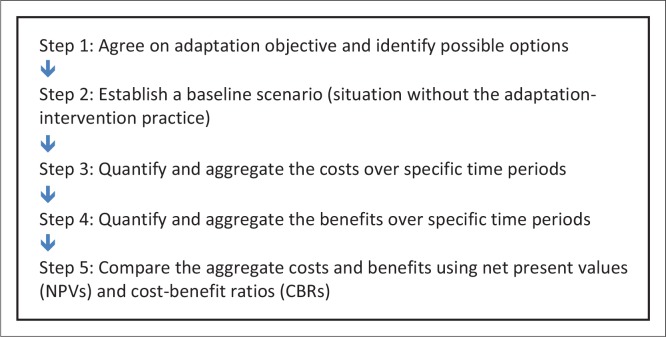
Steps in cost-benefit analysis.

A five-step approach was followed in the CBA (Figure 5).

#### Decision criterion for selecting the optimal adaptation strategy

The benefit-cost ratio (BCR) was used to compare the efficiency of different adaptation options available to farmers from Lambani and Alice. The BCR is an indicator used in CBA that attempts to summarise the overall value for money of a project. The BCR can also be defined as the ratio of benefits, expressed in monetary terms, relative to the costs, also expressed in monetary terms. All benefits and costs are expressed in discounted present values. The BCR takes into account the amount of monetary gain from a project versus the cost of executing the project. The higher the BCR, the higher the return on investment. It is important to note that long-term BCRs, such as those involved in climate change, are very sensitive to the discount rate used in the calculation of net present value, and there is often no consensus on the appropriate rate to use.

### Research design

#### Sample

The study was conducted in the Eastern Cape (Alice) and Limpopo (Lambani). The two provinces were selected because of their vulnerability to disasters and high incidences of poverty and unemployment. The two provinces are mainly rural and highly dependent on rain-fed agriculture. Interviews were carried out with 4040 households in the Eastern Cape (1546) and Limpopo (2494). Respondents were selected based on their willingness to participate in the research. Trained enumerators interviewed the household head after obtaining verbal consent. In the absence of the household head, another adult who stayed at the house on a full-time basis was interviewed.

#### Questionnaire

A structured questionnaire was used to collect data on how climate change was related to various dimensions of household vulnerability such as financial, human, natural, physical and social-capital dimensions. In order to test the validity and reliability of the questions, the HVI questionnaire was pre-tested in both study sites.

#### Data processing, storage and analysis

Prior to analysis, the collected data was cleaned (identifying and removing outliers), and the original questionnaires were revisited to address obvious flaws in data entries and then coded. The data from the HVI questionnaire were then imported from MS Excel into the Statistical Package for Social Sciences (SPSS), version 22.0, for analysis.

## Results

### The impact of climate change on crop systems

The DSSAT model was used to simulate the impact of climate-change scenarios on maize yields in each location. Literature and data collected through site visits were used to determine agronomic conditions, which estimate realistic conditions for farmers in each location (Table 2). All conditions were held constant for each simulation period and scenario, thereby representing only the effect of climatic change on maize yields. The setup consisted of simulations with downscaled climate scenarios, a locally calibrated crop model and locally representative agronomic practices. This allowed for an impact assessment focused on a particular area, which is important since smallholder farmers operate at local scales where conditions (biophysical and socio-economic) vary considerably over short distances, for example, from one district to the other.

**TABLE 2:** Simulated biophysical conditions and agronomic management strategies.

Location	Alice	Lambani
Crop	Maize hybrid: PAN6777	Maize hybrid: SNK2147
Soil	Loam	Clay
Plant density	45 000 plants/ha	44 400 plants/ha
Fertiliser application	Basal: 13 kg N/ha Top: 13 kg N/ha	Basal: 20 kg N/ha Top: 10 kg N/ha
Planting dates	Mid November	Mid November

**FIGURE 6:**
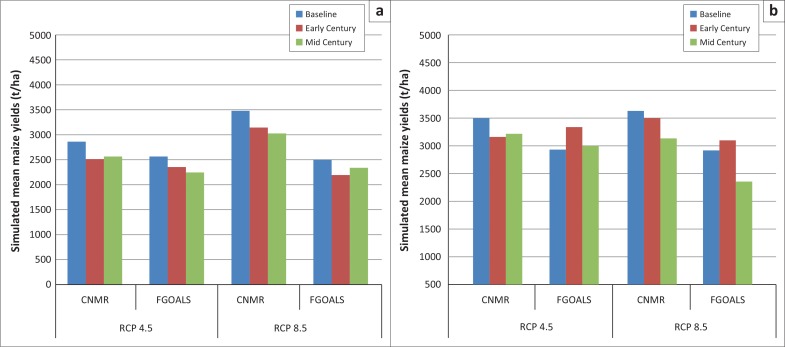
Simulated mean yields per location (a) Alice and (b) Lambani.

The climate projections were used to drive the DSSAT model to simulate maize-yield responses. Figure 6 shows the simulated mean maize yields per location whilst Table 3 shows the simulated mean change and variation in maize yields per location.

**TABLE 3:** Simulated mean maize-yield changes and coefficients of variation.

Variables	Location	Time period	RCP 4.5		RCP 8.5	
			CNRM	FGOALS	CNRM	FGOALS
Mean yield change	Alice	Early century	−15.2	−8.2	−9.6	−13.3
		Mid century	−14.3	−12.4	−13	−6.4
	Lambani	Early century	−9.1	13.7	−3.5	5.9
		Mid century	−8.3	2	−13.9	−19.6
CVs of simulated yields	Alice	Early century	33.2	37.5	33.7	32
		Mid century	37.2	33.7	35.6	39.7
	Lambani	Early century	22.8	21.2	19.6	23.2
		Mid century	26.2	33.6	22.7	27.9

RCP, Representative Concentration Pathways; CNRM, Centre National de Recherches Météorologiques; FGOALS, Flexible Global Ocean-Atmosphere-Land System.

The results show that the impact of climate change, whilst being negative, are location specific. Alice and Lambani are clearly affected differently in the early 21st century. In Alice, there is a strong correlation between the negative impact of climate change and maize yields. However, for Lambani, there is uncertainty about whether the impact of climate change will be negative or positive in the early 21st century (but projections are more negative in the mid-21st century). These results concur with those in other areas in Southern Africa such as the study by Zinyengere *et al*. (2013) that found the projected impact of climate change on crops to be uncertain for the early part of the century but robustly negative further into the 21st century. This points towards different vulnerabilities of maize-production systems to climate change and the need for area-specific interventions. It should be noted that the simulations were made with historical agricultural practices and do not reflect potential benefits from future adaptation and improved technology.

Alice in the Eastern Cape is vulnerable to disasters because of poor physical conditions for farming and an over-dependence on social grants. This is supported by the robust negative impact of climate change on maize production in Alice. The negative impact is reflected even early in the 21st century (2010–2040) where climate variability is expected to be strong, thereby masking the climate-change signal. Regardless, Alice is shown to experience a decline in maize yields. The strong negative impact is expected to persist further into the mid-21st century. These results suggest that the Eastern Cape will require significant support to cushion the province against the adverse effects of climate change.

Lambani in Limpopo Province is also vulnerable to climate change and other natural disasters. The physical conditions in Limpopo present challenges for maize production (low rainfall, high temperatures, high evapotranspiration, poor soil, et cetera). Climate change is likely to increase pressure on food security in the area. Projections for Lambani suggest that, early in the 21st century (2010–2040), it is uncertain how climate change will affect maize yields. Projections from one model suggest a decline in yields whilst the other suggests an increase in yields. This uncertainty may be because climate variability could be stronger than climate change during the period. However, further into the 21st century (2040–2070), climate change is significantly greater than climate variability, and the impact on maize production is robustly negative. Maize production would likely suffer from climate change, but the area’s diverse agricultural activities may provide a buffer to disaster-related crop losses.

### Climate change and mixed crop–livestock systems

Mixed crop-livestock systems enable farmers to integrate different enterprises on the farm: Livestock provide draft power to cultivate the land and manure to fertilise the soil, and crop residue occasionally feed livestock in some areas. Moreover, income from livestock may be able to buffer low crop yields in dry years (Herrero *et al*. 2010). The synergies between crop and livestock rearing offer many opportunities for sustainably increasing production since they can raise productivity and resource use efficiency for farming households. This, in turn, can increase income and secure the availability of and access to food for farming households (Herrero *et al*. 2010).

More than 70% of the resource-poor farmers in South Africa are situated in harsh agro-ecological zones where cropping is marginal. Thus, they rely on livestock or mixed farming for their livelihoods (Bester *et al*. 2003). Limpopo and Eastern Cape fall into this category. Livestock farming is an important agricultural practice in the Eastern Cape, which has the highest percentage of livestock (especially cattle and sheep) compared to the other eight provinces of South Africa (DEDEA 2011). Smallholder livestock farming is quite prominent (Nkonki 2007) with communal areas contributing more than 65% of the livestock (ECDC 2003). In Limpopo, 80% of the farmers practice agriculture on a subsistence basis. Whilst maize production comprises the main component in smallholder farming, more than 50% of available farming units in the province can be allocated to animal husbandry.

In the Eastern Cape and Limpopo, crop production has historically suffered from unfavourable physical conditions (poor soil, poor rainfall, high temperatures, et cetera) and poor management, leading to perennial poor yields. Similarly, insufficient grazing, weak institutional capacity to manage common grazing resources, small herd sizes and livestock diseases have greatly limited the potential for livestock production in the smallholder sector to sustain rural livelihoods (Bayer, Alcock & Gilles 2004). Add to this mix the potential challenges posed by a changing climate, along with possible increases in the occurrence of extreme climatic events (especially droughts), and there is a glaring need to strengthen crop and livestock production against hazards – and so preserve food strategies and livelihoods in these already marginal areas.

Adaptation can come in the form of planting higher-producing or more drought-tolerant crops, the use of higher-potential or more drought-tolerant livestock genotypes and species, moving livestock to more productive pastures, changing the relative emphasis in the farming system on crop-versus-livestock activities and abandoning cropping activities altogether (Jones & Thornton 2009).

Support for crop-livestock farmers should be appropriate to smallholding and multi-purpose production systems and facilitate the development of local market-oriented subsistence production. Intensifying the current production systems could gradually build smallholder farmers’ commercial production capacity. Including integrated systems of livestock and dual-purpose crops could help buffer smallholders from climate-related losses in crop yield while supporting a fledgling livestock sector. Training in pasture-land management, disease control and crop-livestock husbandry and schemes to access input and markets that overcome the disadvantages of smallholder farming communities could also be promoted. Crop production, livestock rearing and other land-based livelihood activities are an important source of employment, food and income for many smallholder farmers. Thus, appropriate policy has the potential to increase these benefits in light of a changing climate. However, it is important to realise that agriculture on its own (crop and livestock production) cannot solve the problems of rural poverty. Where appropriate, other non-farming, low-risk activities could be promoted to support local livelihoods.

### Household vulnerability

The vulnerability of households to climate change is a function of biophysical and socio-economic factors. In this study, the starting point is that vulnerability is conceptualised as a state that exists before encountering a climatic shock (Gbetibouo & Ringler 2009). The analysis focuses on the drivers of the current adaptive capacity and the susceptibility of the household to risks induced by climate change. The current adaptive capacity of households is depicted by their human, physical, financial, natural and social capital. These forms of capital influence their vulnerability. Households can convert the capital from one form to another, depending on their needs and the nature of the shocks. Poor households have little capital and are therefore more vulnerable to the impact of climate change (Eriksen & Silva 2009). Such households often need considerable external assistance to cope with external shocks such as floods and food insecurity.

**FIGURE 7:**
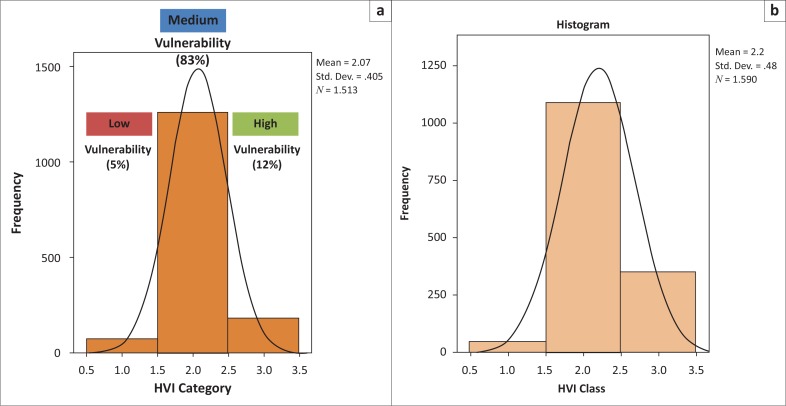
Different vulnerability levels of households from (a) Lambani and (b) Alice.

The HVI found that Lambani contains a higher proportion of highly vulnerable households than Alice (Figure 7). These households constituted 23.7% and 12% of the interviewed households from Lambani and Alice, respectively. Overall, the majority of the population from the two districts were in the moderate-vulnerability category whilst fewer than 5% of the population from the two districts were in the low-vulnerability category.

### Factors influencing the vulnerability of households at Lambani and Alice

#### Demographic factors

Table 4 shows the results of the ordinal regression model for the three HVI categories. It found that an increase in the age of the household head increases the likelihood of the household being classified as moderately or highly vulnerable, with the effect being larger in Lambani than in Alice. Elderly households are more vulnerable because they are not engaged in productive and income-generating activities. Similarly, the sex of the household head was found to be an important factor affecting the vulnerability of households in both Lambani and Alice: The odds of being classified as moderately vulnerable or highly vulnerable are higher for female-headed households in Lambani (0.245) than in Alice.

**TABLE 4:** The factors influencing the vulnerability category of households in Alice and Lambani.

Variable	Alice (*N* = 1513)		Lambani (*N* = 2581)	
	Coefficient	Standard error	Coefficient	Standard error
Age of household head	0.195***	4.60	0.057***	21.96
Sex of household head	0.019***	4.71	0.245**	2.23
Meals per day – children	−0.221***	−2.55	0.092*	1.79
Remittances received	−0.358*	−2.02	0.446	1.56
Access to crop extension service	0.051***	2.75	−0.446	1.25
Receives food support	0.3789*	1.82	−1.454**	−13.52
Knowledge of climate change	−0.587***	−3.72	0.189**	2.15
Training in climate change	0.3298*	1.66	0.054	1.10
Indigenous adaptation	−0.257	−1.55	0.054	1.22
Land ownership	−0.0.72	−0.27	0.093	0.67
Modern adaptation	−0.3269***	−1.90	0.084	1.45
Low yields due to climate change	−0.858***	−4.11	−0.008	−0.10
Low yields due to crop disease or pests	0.00	1.45	0.124	1.18
Increase in food prices	−0.035	−0.23	−0.090	−0.92
Death of family members	0.281	1.23	0.254**	2.37
Formal credit scheme in community	−0.128**	−2.86	−0.054**	−1.90

***, **, *, Statistically significant at 1%, 5% and 10%.

**FIGURE 8:**
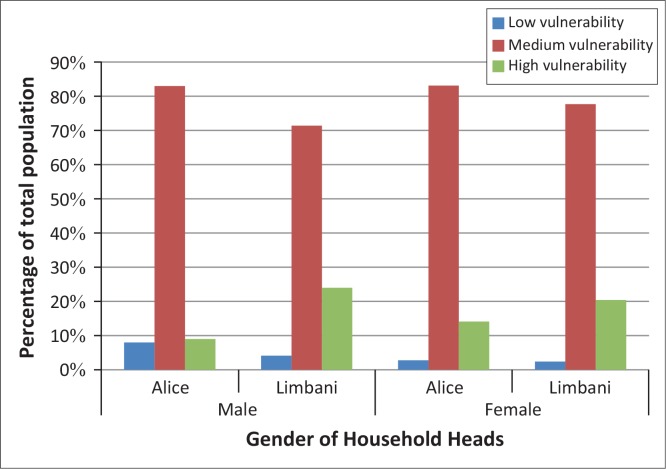
Gender dimensions of vulnerability.

The gender dimensions of vulnerability are further exposed in Figure 8, which reveals that the sex of the household head influences the household’s vulnerability status in different ways based on its location. The proportion of male-headed households regarded as highly vulnerable to climate-induced disasters was significantly greater in Lambani (16%) than in Alice. Surprisingly, in Lambani, of the households regarded as highly vulnerable, 24.4% were headed by males whilst 20.4% were headed by females. Further research is needed to understand the reasons for the higher levels of vulnerability for male-headed households in Lambani.

#### External support for households

Households that receive external food support were found to be more likely to be highly vulnerable in Alice than in Lambani. In Lambani, government is the major source of food aid (3%), followed by family members or friends (2%) and relatives (2%). Although NGOs also provide food aid, their involvement was not so visible. All the households receiving food aid believed that they were poor and thus deserving of it. Other reasons cited were unreliable production systems because of lack of inputs (65%), crop failure (45%) and inadequate labour (21%).

Households also receive external support through remittances. Financial support helps households to deal with shocks to their livelihoods, and so households that receive remittances are more likely to be in the low-vulnerability category. Remittances had no effect on a household’s level of vulnerability in Lambani but decreased the level of vulnerability in Alice. The flow of remittances is often from migrant workers in urban areas to rural areas. Therefore, households living in semi-urban Lambani do not receive substantial remittances, unlike rural households in Alice where the decline in remittances will lead to a decline in farm income, which will affect consumption and hence welfare.

Participation in formal saving schemes in the village is also associated with lower levels of vulnerability. Households that participate in formal savings schemes in the village are more likely to be classified as lowly vulnerable in both Lambani and Alice. Community savings in the village are equally important because of the highly variable income of the poor and the frequency and magnitude of extreme climatic events such as drought and floods. Sustainable and reliable access to savings provides the family with an effective cushion against shocks and allows them to keep their productive physical assets (such as livestock) even in times of crisis.

#### Knowledge of and sensitisation concerning climate change

Households with some knowledge of climate change are less likely to be highly and moderately vulnerable than households with less knowledge. This suggests the need for educational programmes that enhance knowledge on the risks of climate change. In Alice where the majority of households are highly dependent on crop production, households with access to extension services are less likely to be highly or moderately vulnerable. In Lambani, households who implement modern adaptation strategies were more likely to be in the low-vulnerability category.

#### Major shocks experienced by households

**FIGURE 9:**
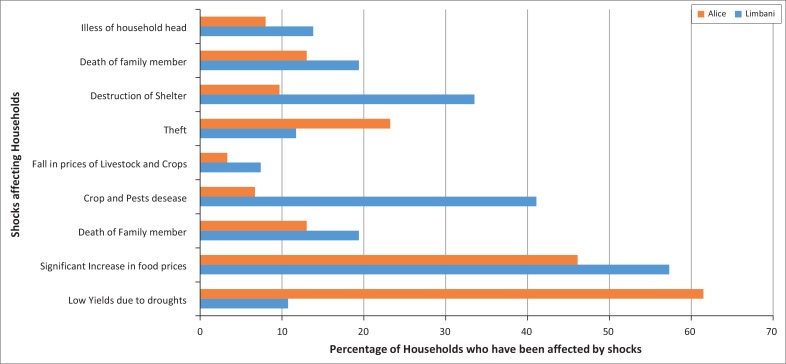
Major household shocks.

Households from Lambani and Alice experienced different shocks concerning their livelihoods and are affected differently, depending on their assets, as Figure 9 shows.

The five major shocks that result in lower yields are droughts, significant increases in food prices, crop diseases or pests, damaged housing and theft. Lower yields due to drought affected 61.5% of households in Alice, compared to 10.7% households in Lambani. Households that produce their own food are more likely to be affected by climate change. Therefore, households in Alice are more vulnerable to climate change than those in Lambani.

Other shocks include loss of employment, the failure of household businesses, non-payment of salary and illness of household members and/or household head. These shocks force households to adopt coping strategies, which deplete the asset endowment of households, reducing the resilience and the capacity of the household to adapt to disasters induced by climate change. The main coping strategy is spending cash savings (18% of all the households studied), followed by reducing food consumption (5%), borrowing money (4%), sending children to live with relatives (4%), relying on external aid (2%), selling assets (1%) and stopping children from going to school (1%). A negligible number of households sold livestock and started new businesses.

### Adaptation strategies: Cost-benefit analysis

CBA’s key strength lies in determining the net economic benefits or costs of different adaptation options, thus assisting the decision makers to allocate resources efficiently in an area. CBA was used to choose the best adaptation strategies from a number of discrete alternative strategies. Table 5 shows the adaptation strategies evaluated in this study.

**TABLE 5:** Adaptation strategies perceived as important in Lambani and Alice.

Adaptation strategy	Brief description
Plant drought-resistant crops (e.g. sorghum)	Unreliable rainfall, changing weather patterns and maize diseases have greatly affected the productivity. This has resulted in low yields and income for farmers. Farmers are now adapting to these changes by shifting from maize to planting drought-resistant crops such as sorghum, finger millet, cow peas and cassava.
Irrigation farming	As the global climate is continuously warming, a significant change in the irrigation of agricultural crops is expected and will result in fewer losses due to climate change and in higher productivity.
Use high-yielding varieties	High-yielding varieties are crops that have been specially selected to produce more than the natural varieties of the same species.
Use organic fertiliser	Organic farming is a form of agriculture that relies on techniques such as crop rotation, green manure, compost and biological pest control. Organic agriculture is an ecological production-management system that promotes and enhances biodiversity, biological cycles and biological activity in the soil. It is based on minimal use of off-farm input and on management practices that restore, maintain and enhance ecological harmony.
Conventional farming system	It is a prevalent form of modern agriculture characterised by the heavy use of synthetic pesticides, fertilisers and machineries. Most conventional farming uses large-scale monocultures of highly selected or pure-bred cultivars. This produces large quantities of food per unit area of cultivated land and per unit of human labour. Many believe that conventional farming has contributed, in a major way, to the continued increase of food production worldwide over the past 60 years.
Zero/minimal tillage farming system	This system encourages soil protection and care through reduced tillage practices and the maintenance of surface residues. It minimises soil disturbance, encourages build-up of organic material, preserves the soil structure and conserves soil water.

Table 6 shows the BCR for different adaptation options in Alice from 2012 to 2042, using a discount rate of 12%. These are the strategies that yield the highest incremental benefit for farmers. They make sense economically as the benefits outweigh the costs (positive net present value).

**TABLE 6:** Benefit-cost ratios for the selected adaptation strategies for Alice.

Rank	Adaptation strategy (BCR)
Sorghum rain-fed	1.93
Zero/minimal tillage maize farming – irrigation	1.50
Sorghum irrigation	1.48
Zero/minimal tillage maize farming – rain-fed	1.44
Maize conventional farming – irrigation	1.37
Maize conventional farming – rain-fed	1.34

BCR, benefit-cost ratio.

Investment in growing drought-resistant crops deserves greater prioritisation in the Eastern Cape, followed by a maize-farming system using zero or minimal tillage. As Table 6 shows, for every rand invested in sorghum, the return on the investment will be R1.93, compared to R1.34 for maize under a rain-fed cropping system. There is substantial potential for increasing farming income through increasing yields by using drought-resistant varieties, conservation farming and a zero-tillage farming system.

For Lambani, 14 adaptation strategies were analysed (Table 7). All but two strategies were found to make sense economically as the benefits outweigh the costs (i.e. have a positive net present value).

**TABLE 7:** Benefit-cost ratios for the selected adaptation strategies for Lambani.

Adaptation strategy	BCR
Sorghum under irrigation	2.06
SNK maize-limited tillage under irrigation-farming system	1.89
Sorghum dry-land farming	1.81
SNK maize conventional farming system, rain-fed, no insurance	1.50
Groundnuts enterprise	1.49
Crop rotation: beans followed by maize	1.49
SNK maize zero/minimal tillage maize farming – rain-fed	1.38
SNK maize conventional irrigation-farming system	1.29
Crop rotation: beans followed by sorghum	1.23
SNK maize conventional rain-fed farming system	1.22
Beans enterprise under irrigation	1.10
Beans enterprise	1.04
Open pollinated varieties and limited tillage – rain-fed	0.93
OPV maize conventional farming system – rain-fed	0.89

BCR, benefit-cost ratio.

The analysis shows that there is substantial potential for increasing farming income by increasing yields through the use of drought-resistant varieties and conservation farming. This is logical, given the productivity and economic benefits of irrigation and conservation farming found around the world (Van Steenbergen & Mehari 2009). Implementing such adaptation strategies would increase both production and productivity per unit area of maize in Limpopo.

## Conclusion

The study evaluated the impact of climate change on agricultural productivity in two rural communities in the Eastern Cape (Alice) and Limpopo (Lambani). The location-specific assessment allowed for the differentiation of local impact. As the first micro-analysis of the impact of climate change, the study contributes to a better understanding of the complex effects of climate change on rural communities. Such understanding will not only highlight the magnitude of the challenge but also improve policy targeting and community-based monitoring.

The impact of a changing climate on maize production^3^ were modelled to assess how food security and livelihoods would be affected. This was achieved by downscaling climate projections, locally setting up a crop model and modelling locally relevant practices. Location-specific data included the climate, soil, crop varieties and agronomic practices (fertiliser applications, planting dates, soil management, tilling, et cetera).

Climate projections for each location clearly showed an increase in temperatures for the early and mid-21st century relative to the baseline with higher temperature increases further into the century. However, rainfall projections were uncertain across all scenarios and locations with no clear indication of whether rainfall will increase or decrease. The DSSAT model was shown to have the ability to simulate maize response to historical climate and agronomic practices in Alice and Lambani. The downscaled climate projections for the future were used to drive the DSSAT crop model to simulate the effects on maize. For Alice, the impact was found to be negative for both the early (−8.2% to −15%) and mid (−6.4% to −14.3%) 21st century. Whilst for Lambani, the impact on the mid-21st century was also strongly negative (+2% to −19%), results revealed uncertainty as to whether maize yields would increase or decrease in the early 21st century (+13.7% to −9.1%). Inter-annual variability in simulated maize yields was found to be higher in Alice than in Lambani.

The results clearly indicate that, by the mid-21st century, maize production will be negatively affected by climate change in both Alice and Lambani. Considering that maize is the stable food crop in both locations and given the prevailing vulnerability owing to unfavourable biophysical conditions and sub-optimal farming practices, climate change presents a concern for households’ food security in these areas. Although the modelled impact for maize production under climate change in Alice and Lambani strongly suggest that maize yields will be negatively affected by the 2050s, it is important to note that simulations are based on current smallholder agricultural practices and therefore represent sub-optimal cropping conditions. Simulations were also made without including the potential effects of future atmospheric carbon dioxide (CO_2_) changes, which along with suitable adaptation strategies could lessen the negative impact of climate change on maize production.

The second objective of this study was to evaluate households’ vulnerability to climate change, a much neglected area. The key issue was to determine which households are vulnerable to disasters related to climate change and why? The study sought to assess how vulnerable the farming systems in Alice and Lambani are to climate change. These communities depend on crop and livestock farming with rural households in Alice practicing less farming than those in Lambani.

The study is grounded in the notion that vulnerability is a function of five types of capital: human, physical, financial, natural and social. Poor households have less capital to liquidate in case of a climate-change shock. In contrast, households with abundant capital can easily convert these to insulate themselves against adverse shocks.

Regarding the question concerning the identity of the vulnerable, the following characterisations of households stood out: Vulnerability increases with age. Vulnerability has gender dimensions. Female-headed households are more vulnerable than male-headed ones. Remittances, participation in formal savings schemes in villages and extension services are key to minimising household vulnerability. Some knowledge of climate change reduces households’ vulnerability. Households with knowledge about climate change tend to adapt to and mitigate the impact of climate change.

Overall, these results suggest that holding assets are important in defining household vulnerability. This implies that a programme that helps to build a household’s asset base would go a long way in strengthening the resilience of households when subjected to external shocks caused by climate change. Government’s social-protection interventions such as the Expanded Public Works Programme, social grants, school-fee assistance and feeding programme programmes at schools are critical and need proper targeting.

As livelihoods in Alice depend more on natural resources and the environment, households in Alice are more directly vulnerable to climate-change disasters than those in Lambani where the impact is largely indirect. Measures to reduce vulnerability to climate-induced disasters should therefore focus on households building sound adaptation strategies and the capacity to adapt to climate change in Alice. Measures to reduce vulnerability to climate-induced disasters in Lambani should focus on improving household resilience, including deepening the extension-services programme that can also act as the main catalyst for sensitising households to climate-change risks and adaptation to climate change. Most households were affected by lower yields due to drought, which reveals a need to integrate early-warning systems and mechanisms in the local-extension service.

The CBA indicates that the use of drought-resistant crop varieties and conservation farming has the potential to increase farm income through increasing yields. Priority should be given to drought-resistant varieties, small grains and zero-tillage farming systems in Limpopo and the Eastern Cape. By adopting zero tillage, smallholder farmers will be practicing climate-smart agriculture which is a combination of adaptation, mitigation and productivity growth. Given that the majority of farmers in the two regions are classified as smallholder farmers, low-cost adaptation options are recommended. These could include crop rotation and sustainable co-benefiting of crops. If done at the local smallholder scale, the irrigation-adaptation option may not be the most prudent because of the required initial-capital investment and maintenance.

## Footnotes

1 Original data obtained from the Computing Centre for Water Research and the South African Weather Service.

2 This was found to be the case for the Strengthening Evidence-based Climate Change Adaptation Policies (SECCAP) FANRPAN project funded by the International Development Research Center (IDRC).

3 Maize is the staple food in both communities. However, it is acknowledged that maize in not the only crop.
